# Bis[2-(2-hydroxy­benzoyl­hydrazono)­propionato]nickel(II) trihydrate. Erratum

**DOI:** 10.1107/S2056989015022690

**Published:** 2016-09-30

**Authors:** Wei-Ping Wu, Feng-Chun Zeng, Yu Wu

**Affiliations:** a Department of Chemistry, Sichuan University of Science and Engineering, Zigong 643000, People’s Republic of China

## Abstract

In the paper by Wu *et al.* [*Acta Cryst.* (2007), E**63**, m2664], the metal atom was reported incorrectly.

## Abstract 2

Erratum to *Acta Cryst.* (2007), E**63**, m2664.

The authors of Wu *et al.* (2007) ▸ have indicated that the metal atom in the reported complex was assigned incorrectly. The paper is therefore withdrawn from the published literature and associated databases.
